# RETRACTION: A Meta‐Analysis of the Risk Factors for Neurosurgical Surgical Site Infection Following Craniotomy

**DOI:** 10.1111/iwj.70520

**Published:** 2025-04-18

## Abstract

**RETRACTION:** SunD. , MaZ. , GengY. , KongC. , and LiZ. , “A Meta‐Analysis of the Risk Factors for Neurosurgical Surgical Site Infection Following Craniotomy,” International Wound Journal 21, no. 4 (2024): e14542, 10.1111/iwj.14542.38140754 PMC10961046

The above article, published online on 22 December 2023, in Wiley Online Library (http://onlinelibrary.wiley.com/), has been retracted by agreement between the journal Editor in Chief, Professor Keith Harding; and John Wiley & Sons Ltd. Following an investigation by the publisher, all parties have concluded that this article was accepted solely on the basis of a compromised peer review process. The editors have therefore decided to retract the article. The authors did not respond to our notice regarding the retraction.

**RETRACTION:** D. Sun , Z. Ma , Y. Geng , C. Kong , and Z. Li , “A Meta‐Analysis of the Risk Factors for Neurosurgical Surgical Site Infection Following Craniotomy,” International Wound Journal 21, no. 4 (2024): e14542, 10.1111/iwj.14542.38140754 PMC10961046

The above article, published online on 22 December 2023, in Wiley Online Library (http://onlinelibrary.wiley.com/), has been retracted by agreement between the journal Editor in Chief, Professor Keith Harding; and John Wiley & Sons Ltd. Following an investigation by the publisher, all parties have concluded that this article was accepted solely on the basis of a compromised peer review process. The editors have therefore decided to retract the article. The authors did not respond to our notice regarding the retraction.

